# Preparation of Low-Fishy Microencapsulated DHA-Rich Algal Oil Powder Using Infant Rice Powder

**DOI:** 10.3390/foods13233827

**Published:** 2024-11-27

**Authors:** Yuqing Zhang, Zuohua Xie, Siqiong Zhang, Jing Li, Ting Luo

**Affiliations:** 1 School of Food, Nanchang University, Nanchang 330000, China; 407900220003@email.ncu.edu.cn; 2 Jiangxi Deshang Technology Group Co., Ltd., Zhangshu 331208, China; xzh10030606@163.com; 3 Jiangxi Guanglai Health Production Co., Ltd., Zhangshu 331208, China; dsyjy2008@163.com

**Keywords:** infant rice powder, DHA-rich algal oil, microencapsulation, relative odor activity value

## Abstract

Commercial DHA-rich algal oil has some issues, such as an unpleasant odor and susceptibility to oxidation. The main fishy odor compounds in commercial DHA-rich algal oil powder and DHA-rich algal oil microcapsules are hexanal and (E, E)-2,4-heptadienal. To address this issue, a microencapsulation process was designed for DHA-rich algal oil using infant rice powder (IRP), maltodextrin (MD), and whey protein concentrate (WPC) as wall materials, with sodium starch octenyl succinate (SSOS) and monoacylglycerol (MAC) as emulsifiers. The spray-drying method was used for microencapsulation. The experimental data showed that microcapsules with wall materials in a ratio of IRP/MD/WPC = 1:3:1 and an emulsifier content of 3.5% (SSOS and MAC) had the highest encapsulation efficiency (85.20 ± 6.03%) and the lowest aldehyde content (65.38 ± 3.23%). This microcapsule showed a good appearance and better oxidation stability compared with the crude oil, with a water content and average particle size of 1.69 ± 0.57% and 631.60 ± 23.19 nm, respectively. The results indicated that DHA-rich algal oil microcapsules prepared with infant rice powder had a lower fishy odor and better sensory acceptability compared to commercial DHA-rich algal oil powder.

## 1. Introduction

DHA is an omega-3 polyunsaturated fatty acid that is essential for the brain, maintaining the growth of nervous system cells and promoting intellectual development in infants and young children [1]. The human body cannot synthesize DHA on its own, and most of it needs to be obtained through food [2]. When breastfeeding is not possible, DHA supplementation through food becomes necessary [3]. Studies have shown that consuming DHA-rich formula can help increase the DHA levels in infants and improve infant development, including cognitive function, vision, and the immune response [4]. Algal oil is the preferred choice for commercially produced DHA powder because it is high in DHA, low in cost, easily available, free of heavy metals, and is considered a vegetarian source of omega-3 fatty acids [5]. Algal oil is the main source of DHA supplementation for infants, and it is currently in 99% of all infant formula sold in the US [6]. The genera *Phaeodactylum*, *Thraustochytrium*, *Nannochloropsis*, and *Schizochytrium* contain abundant amounts of DHA and EPA [7]. When Batista et al. extracted DHA from *I. galbana* using a combined method, the extraction rate reached 1156 mg/100 g [8].

The main obstacle to the application of DHA is its high oxidative sensitivity, which produces oxidation products that may be harmful to humans, and its unpleasant flavor [9]. Therefore, various techniques have been employed to improve the oxidation stability and/or organoleptic acceptability of DHA, among which microencapsulation is a promising technique that has been widely used [10]. Therefore, the DHA-rich algal oil was microencapsulated to enhance its stability and mask the undesirable odor.

Microencapsulation is a technique that uses wall materials to create a barrier between the core and the environment, which shields against odors, improves stability, and extends the shelf life [11]. Spray drying is a common microencapsulation technique used in the food industry, and it has been widely employed to encapsulate oils rich in unsaturated fatty acids because of its low cost, high flexibility, wide availability, and continuous operability [12,13]. Spray-drying microencapsulation generally includes dispersing the core material into the wall material solution to form an emulsion or dispersion, and the emulsion forms atomized droplets to produce microcapsule powder through dehydration and drying [14]. Selecting the appropriate wall material type and composition is an important step in preparing a successful microcapsule system [15].

Some studies have found that using maltodextrin as a wall material for encapsulating DHA-rich algal oil can mask its fishy odor, and others have shown that using rice starch and rice protein as wall materials for oil microencapsulation can improve the oxidative stability of the oil [16,17,18]. However, the degree to which these materials mask the fishy odor of algal oil still presents significant challenges. Furthermore, some researchers believe that the wall materials for microencapsulated fish oil should not only mask the fishy odor but also contribute a distinctive flavor that enhances the overall odor quality [19]. The selection and design of microcapsule wall materials should not only consider factors such as the stability, degradability, mass transfer performance, source, and price of the wall material, but also focus on evaluating the protective and release effects of the wall material on the core material [20]. Infant rice powder (IRP) was generally prepared from rice, the main component is starch, it contains some protein, and itself has the fragrance of rice flour. Maltodextrin (MD) is a common carbohydrate wall material for oil microencapsulation, which has the advantages of good drying performance and a low cost [21]. Whey protein concentrate (WPC) has good emulsification potential and a low cost, and can be mixed with maltodextrin to obtain good emulsions due to its surface-active properties [22]. Helena et al. evaluated the potential of maltodextrin combined with different wall materials (starch, whey protein concentrate, and Arabic gum) to microencapsulate flaxseed oil via spray drying. The results showed that the combination of maltodextrin and whey protein had the best lotion stability and oxidation protection [23]. The purpose of this study was to use IRP, MD, and WPC as wall materials to prepare DHA-rich algal oil microcapsules to mask the fishy smell in DHA-rich algal oil and improve its oxidation stability. In addition, the smell of IRP can help to improve the smell quality of microcapsules.

In this study, algal oil was encapsulated in IRP, MD, and WPC mixtures with a 30% solids concentration and 25% oil using spray drying. The ratios of IRP/MD/WPC mixtures and the content of emulsifiers (SSOS, MAC) were optimized based on the encapsulation efficiency. The peroxide value was measured by using an accelerated oxidation method to investigate the storage stability of DHA-rich algal oil microcapsule powder. The volatile components of commercial DHA-rich algal oil powder and DHA-rich algae oil microencapsulated powder were analyzed via headspace solid-phase microextraction (SPEM) gas chromatography-mass spectrometry (GCMS). The key odors of DHA-rich algal oil, DHA-rich algal oil microcapsules, and commercial DHA-rich algal oil powder were analyzed and compared by calculating their relative odor activity values. The main objective was to develop a low-fishy DHA powder with high sensory acceptance and strong stability which can be used in nutritional rice noodles for infants.

## 2. Materials and Methods

### 2.1. Materials

Docosahexaenoic acid (DHA, triglyceride type, ≥35%) from microalgae was purchased from Shandong Holy Leaf Biotechnology Co., Ltd. (Heze City, China). Infant rice powder and commercial DHA-rich algal oil powder (DHA, triglyceride type, 10%) were a kind gift from Jiangxi Guanglai Health Industry Co., Ltd. (Zhangshu, Jiangxi, China). Maltodextrin (MD, 15 < DE value < 20) was purchased from Henan Yimeike Biotechnology Co., Ltd. (Zhengzhou, Henan, China). Whey protein concentrate (WPC80, ≥99%) was purchased from Fonterra Co-operative Group Ltd. (Auckland, New Zeeland). Sodium starch octyl succinate (SSOS, ≥99%) was purchased from Shanghai Xintai Food Company (Shanghai, China). Monoglycerides (MAC, ≥90%) were purchased from Henan Wanbang Chemical Technology Co., Ltd. (Zhengzhou, Henan, China). All the other reagents were of analytical grade and used without further purification.

### 2.2. Method

#### 2.2.1. Microencapsulation of DHA-Rich Algae Oil Using the Spray Drying Method

The DHA microcapsule powders with a 30% solids concentration and 25% DHA-algae oil were prepared according to Table 1. The preparation method was used according to the method described by Karrar et al. [24] with some modifications. For the microencapsulation of DHA-rich algal oil, in the first step, a primary emulsion was prepared. The wall materials of IRP, MD, and WPC and an emulsifier (SSOS) were added to triple distilled water previously heated to 60 °C and allowed to hydrate for 2 h under mechanical stirring (1500 rpm). In the second step, DHA-rich algal oil was heated to 90 °C and the emulsifier, MAC, was added in the oil phase under stirring. Finally, in the aqueous phase, DHA-rich algal oil was added drop-wise under mechanical stirring at 1500 rpm while maintaining the temperature between 50 and 60 °C. The emulsion was kept under vigorous stirring for at least 2–3 h to ensure the proper encapsulation of oil in the wall materials. The emulsion was homogenized using a homogenizer (JN-10HC, Guangzhou, China) for 7 min before the spray drying process.

**Figure 1:**
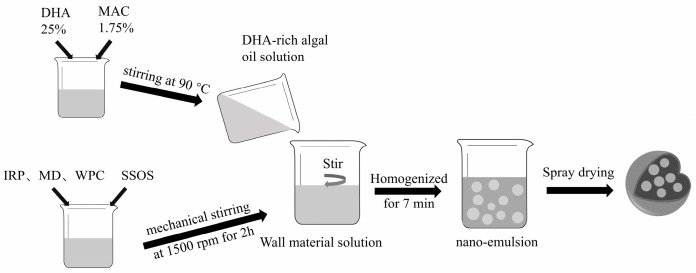
Schematic diagram of microcapsule preparation process.

Spray drying was performed at inlet and outlet temperatures of 180 °C and 80 °C, respectively [25]. The feed pump speed and atomization speed were maintained at 25 mL/min and 50 rpm, respectively. After preparation, DHA powder was collected and immediately stored in 50 mL plastic vials and stored at 4 °C for further analysis within 1 week (Figure 1).

The microcapsules of M1–M10 were prepared via spray drying as follows: microcapsules of M1, M2, M3, M4, and M5 with the ratio of IRP, MD, and WPC of 1:3.2:0.8, 1:3:1, 1:2.4:1.6, 1:2:2, and 1:1.6:2.4, respectively and microcapsules of M6, M7, M8 (M2), M9 and M10 with the emulsifier content of 2%, 2.5%, 3%, 3.5% and 4%, respectively (Table 1).

#### 2.2.2. Characterization of DHA-Rich Algal Oil Microcapsule Powder

The characterization of DHA-rich algal oil microcapsule powder was determined according to the previous method with slight modifications [26,27,28]. The average size, polydispersity index (PDI), and zeta potential of the prepared emulsions of DHA-rich algal oil were measured by using the Malvern Zetasize (ZS90, Malvern Instruments, Malvern, UK). All the microcapsules were prepared in a 0.5 mg/mL solution with deionized water prior to measurement. All the measurements were performed in triplicate. A scanning electron microscopy (SEM) image was used to observe the morphology of the microencapsulated powders via scanning electron microscopy. Double-sided tape was applied to the SEM stage, the microcapsule powder was evenly dispersed on the double-sided tape, and then, the microcapsule stage was sprayed with gold to observe and collect images at 1000× and 2000× magnifications. The moisture content was measured gravimetrically via oven drying at 105 °C until a constant weight was achieved.

#### 2.2.3. Surface Oil Content

The surface oil of the powders was determined using the method described by Zhang et al. [29]. Briefly, 1 g of microcapsule powder was accurately weighed onto filter paper and washed with 5 mL of petroleum ether (BP 40–60 °C) through a funnel. The extract was placed in a pre-weighed glass tube heated in a 60 °C water bath to remove solvents, and then dried to a constant weight in a 105 °C oven to calculate the surface oil content of the microencapsulated product. The amount of surface oil was determined gravimetrically.

#### 2.2.4. Total Oil Content

The methylene chloride-methanol extraction method was used [30]. A total of 4 mL of distilled water was added to 1 g of microcapsules and vortexed for 2 min. Then, the sample was extracted using 5 mL of dichloromethane: methanol (1:2 *v*/*v*) via vortexing for 2 min. Next, the sample was extracted using 5 mL of dichloromethane: water (1:1 *v*/*v*) via vortexing for 2 min. Samples were centrifuged for 10 min at 3000× *g* at room temperature. The organic phase was collected, filtered, and evaporated. The amount of total oil content was determined gravimetrically.

#### 2.2.5. Encapsulation Efficiency

The encapsulation efficiency, which is an approximate measure of the percentage of oil that is not easily removed, was calculated according to the methodology described by Carneiro et al. [23], using the following equation:

Encapsulation efficiency (%) = (Total oil − Surface oil)/Total oil × 100%(1)

#### 2.2.6. Stability Studies

Microcapsules and algal oil were stored at 60 ± 0.5 °C for 12 days to accelerate the oxidation process, and the peroxide value (POV) was measured every 3 days [15,31].

The extraction method of microcapsule oil was as follows: an appropriate amount of microcapsule was weighed into a brown iodine measuring bottle, 0.02 g of papain and 0.02 g of amylase for each 1 g of microcapsule were added, 2 times the sample volume of distilled water was added and mixed well. The iodine measuring flask was placed in a 50 °C constant temperature water bath shaker, and shaking was applied at the rate of 100 times/min for 30 min. After cooling, an equal volume of acetone was added to the sample, mixed evenly, and then, three times the sample volume of petroleum ether was added, shaken, and extracted for 1 min. It was left to stand in a separating funnel for 30 min for layering, and the lower layer was discarded. The organic phase was washed with water equal to the volume of petroleum ether, the lower layer was discarded, and the upper organic phase was transferred to a funnel filled with anhydrous sodium sulfate for filtration. The filtrate was evaporated in a 40 °C water bath to remove the petroleum ether, and the residue was the sample to be tested.

In brief, the extracted oil was dissolved in a 250 mL Erlenmeyer flask, followed by the addition of 30 mL of 3:2 acetic acid/chloroform (*v*/*v*) solution and 1 mL of saturated potassium iodide (KI). After shaking for 1 min, the mixture was kept in the dark for 3 min, diluted with 30 mL of water, and titrated with 0.01 mol/L Na_2_S_2_O_3_ until the yellow color almost disappeared. A starch indicator was added to the mixture, and the titration was continued with the resulting solution until the blue color disappeared. Each test was conducted in triplicate, and a blank test was conducted under the same conditions. The peroxide value was calculated by using the following Equation (2).

Peroxide value (g/100 g) = (V − V_0_) × C × 0.1269/m × 100% (2)

where V = the titration volume of the sample, V_0_ = the titration volume of the blank, C = the concentration of Na_2_S_2_O_3_, and 0.1269 = the mass of iodine equivalent to 1.00 mL of sodium thiosulfate standard titration solution [c (Na_2_S_2_O_3_) = 1.000 mol/L], m= weight of the extracted oil.

#### 2.2.7. Volatile Composition Analysis [32]

A 1.0 g sample was weighed and quickly placed into a 20 mL sample bottle and sealed. After equilibration in a 60 °C water bath for 25 min, a 65 μm PDMS-DVB fiber was inserted at 60 °C for 30 min for solid-phase microextraction; then, the volatiles were directly desorbed in the GC injection mouth for 5 min.

Analysis using GC/MS was performed using a GC mass spectrometer (Agilent 19091S-433UI mass detector). The GC-MS system was equipped with a HP-5ms column (30 m × 0.25 mm inner diameter, 0.25 μm film thickness). The GC oven temperature was initially maintained at 40 °C for 3 min, then increased by 6 °C/min to 200 °C, and finally increased by 10 °C/min to 250 °C for 4 min. The solvent delay was 3 min, and helium was used as a gas carrier at a constant column flow rate of 2 mL/min. The mass spectrometer was operated at 70 eV with a mass range from 30 to 400 amu. The interface temperature was 230 °C. Using the C_10_–C_25_ n-alkane standard as a reference, the retention time of each volatile was converted into a retention index. Volatile compounds were identified by matching the mass spectra to the reference compound spectra in the NIST2014 mass spectral library. The relative percentage content of the experimental compounds was calculated by using the peak area normalization method.

#### 2.2.8. Sensory Analysis [19]

A total of 14 evaluators took part in this experiment. The panelists were instructed to gently rotate the lid open, sniff the headspace of the sample with a short kiss, and tighten the lid at the end of the sniff. The panelists cleared the samples between their nasal passages during a 1 min break. The intensity of the odor attributes was assessed by means of an intensity scale (0–5). The intensity scale scores are expressed as follows: 0 = not detectable, 1 = very slightly detectable, 2 = slightly detectable, 3 = fairly detectable, 4 = strongly detectable, and 5 = very strongly detectable.

Triangular test: six sets of samples were provided, each with two identical samples and one different sample. Each sample was encoded with three random irregular samples, attempting to provide samples in all the possible orders, and the evaluators were required to conduct differential evaluations of the samples.

#### 2.2.9. Calculation of the Relative Odor Activity Value (ROAV)

By calculating the ROAV of the volatile components, the main contributors to the fishy smell of DHA-rich algal oil were determined. The ROAV of the volatile composition was determined according to the methodology described by Zhu et al. [33], using the following equation:

ROAV ≈ 100 × (C/C_stan_) × (T_stan_/T) (3)

where C_stan_ is the relative percentage (%) of the ingredient that contributes the most to the overall flavor level, T_stan_ is its sensory threshold (μg/kg) in water, C is the concentration of the compound, and T is its detection odor threshold in water.

#### 2.2.10. Statistical Analysis

All data are expressed as the mean ± SD, and all the experiments were conducted thrice independently. ANOVA and the independent samples *t*-test with IBM SPSS software version 26.0 was used to determine significant differences (*p* < 0.05).

## 3. Results and Discussion

### 3.1. Characterization of DHA-Rich Algal Oil Microcapsules

The application of microcapsules in food products is largely influenced by the quality and appearance of the produced powder, which are directly dependent on the particle size of the microcapsules [34]. As shown in Table 2, the average particle size and zeta potential changed significantly with the change in wall material and emulsifier, and the effect of the wall material on the particle size potential was greater than that of the emulsifier. The particle size of the DHA microcapsules was about 0.6–1.0 μm, which is similar to the results reported by Kwamman [35], indicating that our substance was successfully encapsulated. In addition, a larger absolute value of the zeta potential, which indicates a strong interaction force between the particles and a strong stability of the particles in the solution [36]. The potential of all the DHA microcapsules was negative, ranging from −35.97 to −24.63 mV, indicating that the method could improve the stability of the DHA microcapsules in an aqueous solution. The wall material and emulsifier had little effect on the PDI of the microcapsules (Table 2).

The moisture content of the microcapsules obtained at different ratios of IRP, MD, and WPC and different emulsifier content is presented in Table 2. The water content of the microencapsulated fish oil is an important parameter because high water activity enhances lipid oxidation [37]. Food microcapsules with a moisture content of 4–6% can meet the requirements for long-term storage [38]. The moisture content of the microcapsules ranged from 1.48 ± 0.04% to 4.93 ± 0.29%, all less than 6%, which is considered to be the ideal characteristics of the powder. This is expected to minimize the opportunity for microbial contamination and lipid oxidation [39].

**Figure 2:**
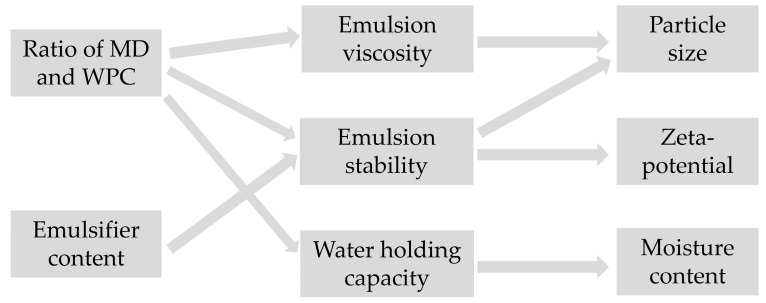
The influence factors of particle size, zeta potential, and moisture content.

Compared with polysaccharide types, protein-based modified hydrophilic colloids have a higher emulsifying capacity (Such as whey protein concentrate, WPC) [40]. Increasing the content of WPC improves the emulsibility of the wall material, thus increasing the stability of the emulsion. The stability of lotion significantly affects its particle size and potential. WPC has a high water holding capacity, and it increases the moisture content, but using MD at the same time can reduce the moisture content [41]. Protein will increase the viscosity of a lotion due to its water-binding and water-retention properties [42]. Therefore, increasing the content of WPC will increase the viscosity of a lotion, leading to an increase in the particle size and water content (Figure 2). Maltodextrin polysaccharides establish hydrogen bonds and electrostatic forces with WPC, thereby improving the protective effect and stability of wall materials [43].

**Figure 3:**
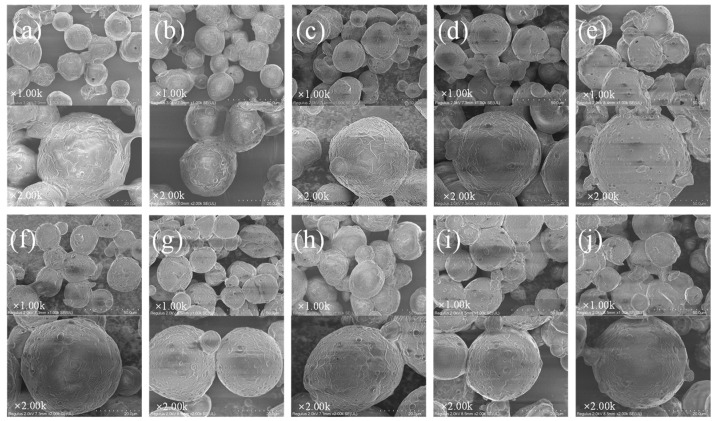
Scanning electron microscopy and photographic images of the spray-dried DHA microcapsules (1.00 K× and 2.00 K× magnification), (**a**–**j**) M1–M10 (M1, M2, M3, M4, M5: microcapsules with the ratio of IRN, MD, and WPC of 1:3.2:0.8, 1:3:1, 1:2.4:1.6, 1:2:2, and 1:1.6:2.4, respectively. M6, M7, M8, M9, M10: microcapsules with emulsifier content of 2%, 2.5%, 3%, 3.5%, and 4%, respectively).

SEM micrographs of the spray-dried powders are shown in Figure 3. All the microcapsules were structurally intact with no visible cracks and well defined edges, most of which were present in a spherical shape. This is potentially beneficial as it means that the microcapsules protected the DHA from oxidation, which is important for the shelf-life stability of the oil microcapsules.

### 3.2. Surface Oil and the Encapsulation Efficiency

**Figure 4:**
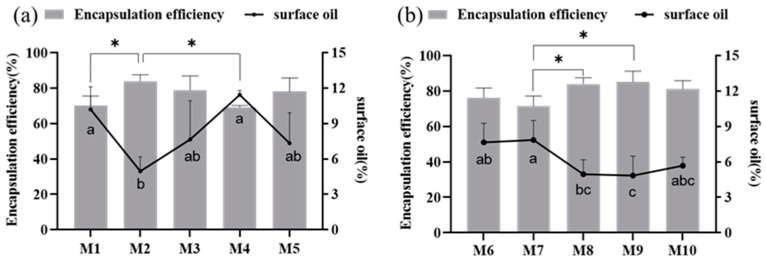
Efficiency of the microcapsules: (**a**) microcapsules with different ratios of IRN/MD/WPC. M1, M2, M3, M4, M5: microcapsules with the ratio of IRN, MD, and WPC of 1:3.2:0.8, 1:3:1, 1:2.4:1.6, 1:2:2, and 1:1.6:2.4, respectively. (**b**) Microcapsules with different emulsifier content. M6, M7, M8, M9, M10: microcapsules with emulsifier content of 2%, 2.5%, 3%, 3.5%, and 4%, respectively. Different letters indicate the significant difference among the samples at *p* < 0.05. * *p* < 0.05.

The encapsulation efficiency (EE) is an important parameter to evaluate the efficiency of the drying process and to define the degree of oil retention inside the wall material [44]. The oil on the surface of microcapsules can affect the encapsulation efficiency and make it susceptible to oxidation, produce odors, affect the acceptability of the product, and lead to a decrease in the powder quality (Figure 4) [39]. As can be seen from Figure 4, the surface oil content of microcapsule M1 to M10 ranges from 4.85 ± 1.64% to 11.43 ± 0.37%, and the EE content ranges from 69.17 ± 1.03% to 85.20 ± 6.03%. The surface oil content and encapsulation efficiency of microcapsules with different wall material ratios and emulsifier content are significantly different, indicating that the IRP/MD/WPC ratio and emulsifier content have a direct effect on the surface oil content (or EE value). The results show that when the solid content was 30%, the DHA-rich algal oil was 25%, the wall material had the IRP/MD/WPC ratio of 1:3:1, and the emulsifier was 3.5% (SSOS, MAC); the microcapsule had the highest EE of 85.20 ± 6.03% and the lowest surface oil content of 4.85 ± 1.64%. Therefore, this condition is the optimal condition for preparing DHA-rich algal oil microcapsules (Figure 4).

### 3.3. Storage Stability

**Figure 5:**
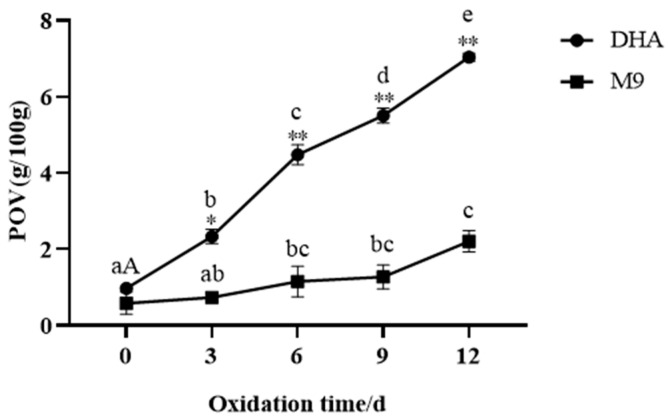
The POV of algal oil and the spray-dried DHA microcapsules. DHA: DHA-rich algal oil; M9: prepared DHA-rich algal oil powder with IRN/MD/WPC = 1:3:1 and 3.5% emulsifier (SSOS and MAC). Different lowercase letters indicate the significant difference among the DHA samples at *p* < 0.05. Different capital letters indicate the significant difference among the M9 samples at *p* < 0.05. * *p* < 0.05, and ** *p* < 0.01.

The POV is a standard assay for assessing the fish oil oxidation after microencapsulation and during storage [45]. The POV is attributed to the increased formation of hydroperoxides, which are the main oxidation products reflecting the oxidative deterioration of oils [46]. Mixtures of MD and WPI have been reported to have excellent protective effects against lipid oxidation [23]. In addition to the wall material composition, surface oil is another factor that affects the oxidative stability [47]. The results are shown in Figure 5, and the initial POV of all the oil samples was ~1.2 g/100 g oil on day 0 prior to storage. The experimental results show that no significant difference was observed between the encapsulated oil and the control oil on day 0. This means that no lipid oxidation occurred during the encapsulation process (emulsion preparation and spray drying). When stored at 60 °C for 12 days, the maximum peroxide value of the DHA-rich algal oil was 7.04 g/100 g (Figure 5), while the peroxide value of the microcapsule products was about 2.20 g/100 g, indicating that the microcapsule products have efficient heat resistance and can improve the oxidative stability of DHA-rich algal oil. The relatively low surface oil content in M9 may be one of the reasons for their improved oxidative stability.

### 3.4. Fishy Flavor Evaluation

#### 3.4.1. Volatile Composition Analysis

**Figure 6:**
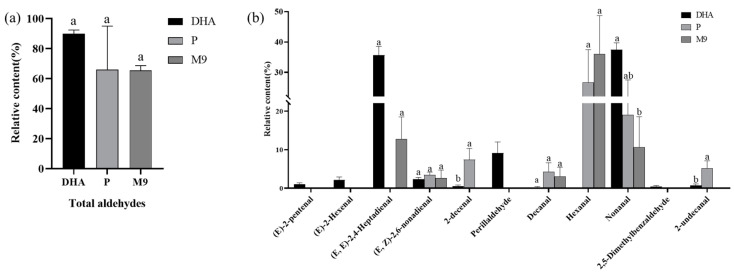
(**a**) The relative content per g of DHA of the total aldehydes of three samples; (**b**) the relative content per g of DHA of the single aldehydes of three samples. DHA: DHA-rich algal oil, 35% DHA; P: commercial DHA-rich algal oil powder, 10% DHA; M9: prepared DHA-rich algal oil powder with IRN/MD/WPC = 1:3:1 and 3.5% emulsifier (SSOS and MAC), 8.7% DHA. Different lowercase letters indicate significant difference at *p* < 0.05 among DHA groups.

The relative content of the characteristic volatile aldehydes of the DHA-rich algal oil microcapsules are shown in Table 3 and Table 4. The relative content of the aldehydes in M9 was the lowest, which was 5.99%. Combined with the results of the encapsulation efficiency and volatile component analysis, the results show that M9 had the highest encapsulation efficiency and the lowest volatile aldehyde content. M9 was selected for comparison with commercial DHA-rich algal oil powder (P) for the study of the key fishy odor components. It can be demonstrated that a specific combination of volatiles imparts a fishy odor, with 1-penten-3-one and (E, Z)-2,6-nonadienal being the most important volatiles contributing to the fishy and metallic taste [48]. Aldehydes are the main fishy substances in fish oils, and the general oxidized, rancid, and lacquered odors in fish oils are caused by saturated and unsaturated aldehydes, including hexanal, (E, Z)- and (E, E)-2,4-heptadienal, and (E, E)- and (E, Z)-2,4-decadienal [49]. The relative content of aldehydes of DHA and P as well as M9 is shown in Figure 6. The total relative content of aldehyde of M9 (65.38 ± 3.23%) is significantly lower than that of DHA (89.93 ± 2.41%, *p* <0.01) and lower than P (66 ± 28.91%) in Figure 6a. In Figure 6b, three common components are shown detected in DHA, P, and M9, which are (E, Z)-2,6-nonadienal, Decanal, and Nonanal, respectively. Compared with DHA, the relative content of (E, E)-2, 4-heptadienal and Nonanal of M9 was significantly reduced. The relative content of (E, Z)-2,6-nonadienal, Decanal, and Nonanal of M9 was lower than that of *p*. (E, E)-2,4-heptadienal is the main characteristic fishy substance in DHA and M9. The results show that the content of fishy odor substances in microcapsule M9 was significantly lower than that of DHA-rich algal oil and lower than commercial DHA-rich algal oil powder.

#### 3.4.2. Sensory Evaluation

**Figure 7:**
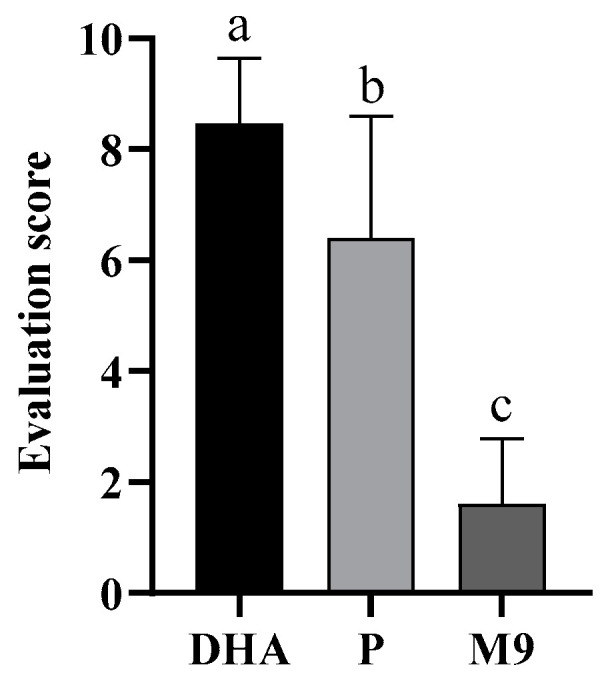
The odor sensory score of the samples. DHA: DHA-rich algal oil, 35% DHA; P: commercial DHA-rich algal oil powder, 10% DHA; M9: prepared DHA-rich algal oil powder with IRN/MD/WPC = 1:3:1 and 3.5% emulsifier (SSOS and MAC), 8.7% DHA. Different letters indicate significant difference at *p* < 0.05 among DHA groups.

The sensory evaluation of the three samples is shown in Figure 7, with M9 scoring the lowest, P the medium, and DHA the highest (average scores of 3.82, 6.39, and 8.46, respectively). It was known that the higher the score, the more fishy the smell and the lower the sensory acceptability. The overall sensory evaluation score of DHA algal oil microcapsules is significantly better than that of DHA crude oil and commercial DHA-rich algal oil powder in terms of odor (*p* < 0.05). Ogrodowska compared the sensory quality of capsules prepared using different coating materials (maltodextrin, whey, sunflower seed and rice protein, and guar gum) and found that the mixed oil encapsulated with rice protein had the lowest intensity of fishy odor [50]. The specific combination of aldehydes forms a fishy odor, while microcapsule encapsulation reduces the relative content of total aldehydes, resulting in a higher sensory acceptance than DHA. The sensory score of microencapsulated DHA algal oil is better than that of commercial DHA-rich algal oil powder. It may be that the addition of infant rice powder as the wall material having its own flavor can also improve the fishy smell. The infant rice powder in the microcapsule can not only be used as a wall material to cover up the fishy smell, but its own aroma can also play a role in improving the fishy smell. This shows that infant rice powder has a good potential as a wall material, which can not only be used as a wall material to cover the fishy smell, but also, its own aroma can play a role in improving the fishy smell.

#### 3.4.3. The Odor Characteristics, Thresholds, and ROAV of Crucial Aldehydes

By calculating the ROAV of volatile flavor compounds, the key flavor compounds of DHA, DHA microcapsules, and commercial DHA powder were identified, and their fishy odor characteristics were described. All the fishy flavor components have ROAVs ranging from 0 to 100, and it is generally believed that the higher the ROAV was, the more it contributes to the overall flavor [51]. When the ROAV ≥ 1, the group was identified as a key flavor substance; when 0.1 ≤ the ROAV < 1, the component also had a certain influence on the overall flavor [52].

The odor characteristics, odor thresholds [53], and ROAVs of the volatile components are shown in Table 5. In DHA-rich algal oil, there are six substances with a ROAV > 1, namely (E, E)-2,4-Heptadienal, Perillaldehyde, (E, Z)-2,6-nonadienal, (E)-2-Hexenal, Nonanal, and 2-decenal. Within the DHA-rich algal oil microcapsules, four substances exhibited an ROAV > 1, specifically (E, E)-2,4-heptadienal and hexanal, (E, Z)-2,6-nonadienal, and nonadienal. This indicates that these components are the key substances that produce a fishy odor. Among DHA and DHA microcapsules, (E, E)-2,4-Heptadienal had the highest ROAV and contributed more to the fishy taste, and had a fishy and grassy taste which is the key substance of the fishy taste of DHA-rich algal oil and its microcapsules. In commercial DHA-rich algal oil powder, the ROAV of hexanal and nonanal is greater than 1, indicating that these two components are the key to the fishy odor. Hexanal has the highest ROAV in commercial DHA-rich algal oil powder, and has a fishy and grassy taste, which is a key substance in the fishy taste of commercial DHA-rich algal oil powder.

## 4. Conclusions

This study successfully developed a low-fishy-odor DHA-rich algal oil powder using infant rice powder as the wall material, which improved its stability and minimized the fishy odor. The wall material of microcapsules has a greater impact on the embedding effect than emulsifiers. The relative odor activity value (ROAV) was used to analyze the fishy odor characteristic substances, and it was found that the key fishy odor substance of DHA-rich algal oil and its microcapsules is (E, E)-2,4-heptadieenal, while the key fishy odor substance of commercial DHA-rich algal oil powder is hexanal. The results demonstrate that DHA microcapsules made with infant rice noodle, maltodextrin, and whey protein concentrate as wall materials effectively mask the fishy smell of DHA-rich algal oil, outperforming commercial DHA-rich algal oil powder. These findings offer valuable insights for DHA fortification in infant rice powder, making it a useful tool for the development of infant food products fortified with DHA.

## Figures and Tables

**Table 1:** Description of formulation.

Treatment Number	Core Content (%)	Wall Materials (IRP/MD/WPC)	Emulsifier (%)(SSOS: MAC = 1:1)	Solid Content (%)
M1	25	1:3.2:0.8	3	30
M2	25	1:3:1	3	30
M3	25	1:2.4:1.6	3	30
M4	25	1:2:2	3	30
M5	25	1:1.6:2.4	3	30
M6	25	1:3:1	2	30
M7	25	1:3:1	2.5	30
M8 (M2)	25	1:3:1	3	30
M9	25	1:3:1	3.5	30
M10	25	1:3:1	4	30

Abbreviations: IRP, infant rice powder; MD, maltodextrin; WPC, whey protein concentrate; SSOS, sodium starch octenyl succinate; MAC, monoacylglyceride.

**Table 2:** Particle size, polymer dispersity index (PDI), zeta potential, and moisture content of the microcapsules.

Samples	Particle Size (nm)	PDI	Ζeta Potential (mV)	Moisture Content
M1	1016.50 ± 12.02 ^a^	0.545 ± 0.328 ^a^	−24.63 ± 0.78 ^a^	4.93 ± 0.29% ^a^
M2	615.55 ± 3.46 ^e^	0.463 ± 0.179 ^a^	−35.97 ± 0.85 ^g^	2.5 ± 0.18% ^cde^
M3	688.95 ± 46.74 ^cd^	0.643 ± 0.347 ^a^	−31.43 ± 0.76 ^de^	1.48 ± 0.04% ^de^
M4	802.20 ± 19.94 ^b^	0.442 ± 0.279 ^a^	−30.27 ± 1.08 ^cd^	1.74 ± 0.01% ^de^
M5	632.40 ± 20.40 ^cde^	0.480 ± 0.029 ^a^	−28.03 ± 0.47 ^b^	2.99 ± 0.44% ^bcd^
M6	734.75 ± 51.97 ^bc^	0.542 ± 0.214 ^a^	−29.27 ± 0.4 ^bc^	3.27 ± 0.98% ^bc^
M7	717.5 ± 13.86 ^c^	0.344 ± 0.303 ^a^	−32.57 ± 1.19 ^ef^	2.02 ± 0.46% ^cde^
M8 (M2)	615.55 ± 3.46 ^e^	0.463 ± 0.179 ^a^	−35.97 ± 0.85 ^g^	2.5 ± 0.18% ^cde^
M9	631.60 ± 23.19 ^de^	0.450 ± 0.047 ^a^	−33.40 ± 0.69 ^f^	1.69 ± 0.57% ^de^
M10	597.75 ± 23.22 ^e^	0.462 ± 0.361 ^a^	−31.50 ± 0.61 ^de^	4.22 ± 1.39% ^ab^

M1, M2, M3, M4, M5: microcapsules with the ratio of IRN, MD, and WPC of 1:3.2:0.8, 1:3:1, 1:2.4:1.6, 1:2:2, and 1:1.6:2.4, respectively. M6, M7, M8, M9, M10: microcapsules with emulsifier content of 2%, 2.5%, 3%, 3.5%, and 4%, respectively. Different letters mean significant differences between the samples within the column (*p* < 0.05).

**Table 3:** The volatiles’ content of different ratios of wall materials to all of microencapsulated DHA algal oils (relative content/g sample).

Aldehydes	M1	M2	M3	M4	M5
(E)-2-pentenal	1.60%	ND	1.01%	1.34%	ND
(E, E)-2,4-Heptadienal	16.88%	2.51%	2.43%	14.25%	3.53%
(E, Z)-2,6-nonadienal	0.76%	1.19%	ND	ND	2.15%
2-decenal	2.61%	0.94%	2.51%	2.24%	1.89%
Hexanal	8.39%	1.38%	0.48%	3.17%	1.75%
Nonanal	7.08%	3.03%	4.56%	6.42%	1.57%
2-Undecenal	1.57%	ND	1.62%	1.71%	0.94%
total	38.90%	9.04%	12.61%	29.12%	11.83%

M1, M2, M3, M4, M5: microcapsules with the ratio of IRN, MD, and WPC of 1:3.2:0.8, 1:3:1, 1:2.4:1.6, 1:2:2, and 1:1.6:2.4, respectively. ND: this aldehyde was not detected.

**Table 4:** The volatiles’ content of different emulsifier content of microencapsulated DHA algal oils (relative content/g sample).

Aldehydes	M6	M7	M8 (M3)	M9	M10
(E, E)-2,4-Heptadienal	1.13%	2.80%	2.51%	1.11%	1.32%
(E, Z)-2,6-nonadienal	0.18%	0.20%	1.19%	0.23%	0.18%
2-decenal	0.13%	ND	0.94%	ND	0.13%
Decyl aldehyde	0.13%	0.11%	ND	0.27%	0.16%
Hexanal	3.00%	4.29%	1.38%	3.14%	3.05%
Nonanal	1.79%	1.17%	3.03%	0.93%	1.54%
2,5-Dimethylbenzaldehyde	0.04%	ND	ND	ND	0.15%
2-Undecenal	0.21%	0.09%	ND	ND	0.06%
total	6.60%	8.66%	9.04%	5.69%	6.60%

M6, M7, M8, M9, M10: microcapsules with emulsifier content of 2%, 2.5%, 3%, 3.5%, and 4%, respectively. ND: this aldehyde was not detected.

**Table 5:** The odor characteristics, taste, and odor thresholds (g/kg of characteristic aldehydes). DHA: DHA-rich algal oil, 35% DHA; P: commercial DHA-rich algal oil powder, 10% DHA; M9: prepared DHA-rich algal oil powder with IRN/MD/WPC = 1:3:1 and 3.5% emulsifier (SSOS and MAC), 8.7% DHA.

Aldehydes	Odor Characteristics	Threshold	ROAV		
			DHA	P	M9
(E)-2-pentenal	Spicy green fruity apple orange tomatoes	1.4	0.12	0.00	0.00
(E)-2-Hexenal	Green banana aldehyde cheese	0.03	11.41	0.00	0.00
(E, E)-2,4-Heptadienal	Fishy, grassy	0.057	100.00	18.26	100.00
(E, Z)-2,6-nonadienal	Green fat dried cucumber purple leaves	0.022	17.42	100.00	60.67
2-decenal	Fat orange, rose, aldehyde, floral, green	3.22	0.03	0.00	0.00
Perillaldehyde	Peppermint smell	0.03	48.68	0.00	0.00
Decanal	Sweet, waxy, floral	3	0.02	0.43	0.54
Hexanal	Fishy, grassy	0.12	0.00	66.15	51.11
Nonanal	Fatty and fragrant	1	5.99	5.65	4.65
2,5-Dimethylbenzaldehyde	ND	0.2	0.37	0.00	0.00
Undecanal	Fatty, sweet orange	5	0.02	0.31	0.00

ND: this aldehyde’s odor characteristics are not found.

## Data Availability

The raw data supporting the conclusions of this article will be made available by the authors on request.

## References

[1] Sun G.Y., Simonyi A., Fritsche K.L., Chuang D.Y., Hannink M., Gu Z., Greenlief C.M., Yao J.K., Lee J.C., Beversdorf D.Q. (2018). Docosahexaenoic acid (DHA): An essential nutrient and a nutraceutical for brain health and diseases. Prostaglandins Leukot. Essent. Fat. Acids.

[2] Innes J.K., Calder P.C. (2018). Omega-6 fatty acids and inflammation. Prostaglandins Leukot. Essent. Fat. Acids.

[3] Marques M.C., Perina N.P., Mosquera E.M.B., Tome T.M., Lazarini T., Mariutti L.R.B. (2021). DHA bioaccessibility in infant formulas and preschool children milks. Food Res. Int..

[4] Lien E.L., Richard C., Hoffman D.R. (2018). DHA and ARA addition to infant formula: Current status and future research directions. Prostaglandins Leukot. Essent. Fat. Acids.

[5] Winwood R. (2013). 14–Algal Oil as a Source of Omega-3 Fatty Acids. Woodhead Publishing Series in Food Science, Technology and Nutrition.

[6] Kuratko C.N., Salem N. (2013). Docosahexaenoic acid from algal oil. Eur. J. Lipid Sci. Technol..

[7] Li X., Liu J., Chen G., Zhang J., Wang C., Liu B. (2019). Extraction and purification of eicosapentaenoic acid and docosahexaenoic acid from microalgae: A critical review. Algal Res..

[8] Batista A.P., Gouveia L., Bandarra N.M., Franco J.M., Raymundo A. (2013). Comparison of microalgal biomass profiles as novel functional ingredient for food products. Algal Res..

[9] Hu Z., Wu P., Wang L., Wu Z., Chen X.D. (2022). Exploring in vitro release and digestion of commercial DHA microcapsules from algae oil and tuna oil with whey protein and casein as wall materials. Food Funct..

[10] Li R., Shi Y. (2017). Microencapsulation of borage oil with blends of milk protein, β-glucan and maltodextrin through spray drying: Physicochemical characteristics and stability of the microcapsules. J. Sci. Food Agric..

[11] Yildiz G., Ding J., Gaur S., Andrade J., Engeseth N.E., Feng H. (2018). Microencapsulation of docosahexaenoic acid (DHA) with four wall materials including pea protein-modified starch complex. Int. J. Biol. Macromol..

[12] Pereira A.R.L., Cattelan M.G., Nicoletti V.R. (2019). Microencapsulation of pink pepper essential oil: Properties of spray-dried pectin/SPI double-layer versus SPI single-layer stabilized emulsions. Colloids Surf. A Physicochem. Eng. Asp..

[13] Loi C.C., Eyres G.T., Silcock P., Birch E.J. (2020). Preparation and characterisation of a novel emulsifier system based on glycerol monooleate by spray-drying. J. Food Eng..

[14] Assadpour E., Jafari S.M. (2019). Advances in Spray-Drying Encapsulation of Food Bioactive Ingredients: From Microcapsules to Nanocapsules. Annu. Rev. Food Sci. Technol..

[15] Chen W., Wang H., Zhang K., Gao F., Chen S., Li D. (2016). Physicochemical Properties and Storage Stability of Microencapsulated DHA-Rich Oil with Different Wall Materials. Appl. Biochem. Biotechnol..

[16] Kurek M.A., Pratap-Singh A. (2020). Plant-Based (Hemp, Pea and Rice) Protein-Maltodextrin Combinations as Wall Material for Spray-Drying Microencapsulation of Hempseed (*Cannabis sativa*) Oil. Foods.

[17] Márquez-Gómez M., Galicia-García T., Márquez-Meléndez R., Ruiz-Gutiérrez M., Quintero-Ramos A. (2018). Spray-dried microencapsulation of orange essential oil using modified rice starch as wall material. J. Food. Process. Preserv..

[18] Jónsdóttir R., Bragadóttir M., Arnarson G. (2005). Oxidatively Derived Volatile Compounds in Microencapsulated Fish Oil Monitored by Solid-phase Microextraction (SPME). Jounal Food Sci..

[19] Serfert Y., Drusch S., Schwarz K. (2010). Sensory odour profiling and lipid oxidation status of fish oil and microencapsulated fish oil. Food Chem..

[20] Jafari S.M., He Y., Bhandari B. (2007). Encapsulation of Nanoparticles of d-Limonene by Spray Drying: Role of Emulsifiers and Emulsifying Techniques. Dry. Technol. Int. J..

[21] Yazicioglu B., Sahin S., Sumnu G. (2014). Microencapsulation of wheat germ oil. J. Food. Sci. Technol..

[22] Pérez-Masiá R., López-Nicolás R., Periago M.J., Ros G., Lagaron J.M., López-Rubio A. (2015). Encapsulation of folic acid in food hydrocolloids through nanospray drying and electrospraying for nutraceutical applications. Food Chem..

[23] Carneiro H.C., Tonon R.V., Grosso C.R., Hubinger M.D. (2013). Encapsulation efficiency and oxidative stability of flaxseed oil microencapsulated by spray drying using different combinations of wall materials. J. Food Eng..

[24] Karrar E., Mahdi A.A., Sheth S., Ahmed I.A.M., Manzoor M.F., Wei W., Wang X. (2020). Effect of Maltodextrin Combination with Gum Arabic and Whey Protein Isolate on the Microencapsulation of Gurum Seed Oil Using a Spray-Drying Method. Int. J. Biol. Macromol..

[25] Chen L., Liu X., Li D., Chen W., Zhang K., Chen S. (2016). Preparation of stable microcapsules from disrupted cell of *Haematococcus pluvialis* by spray drying. Int. J. Food Sci. Technol..

[26] Quỳnh N.T., Hải T.C., Thanh L. (2016). Effect of Wall Material on the Property of Gac Oil Spray-dried Power. J. Nutr. Food Sci..

[27] Fioramonti S.A., Stepanic E.M., Tibaldo A.M., Pavon Y.L., Santiago L.G. (2019). Spray dried flaxseed oil powdered microcapsules obtained using milk whey proteins-alginate double layer emulsions. Food. Res. Int..

[28] Zhu Y., Peng Y., Wen J., Quek S.Y. (2021). A Comparison of Microfluidic-Jet Spray Drying, Two-Fluid Nozzle Spray Drying, and Freeze-Drying for Co-Encapsulating β-Carotene, Lutein, Zeaxanthin, and Fish Oil. Foods.

[29] Zhang Y., Pang X., Zhang S., Liu L., Ma C., Lu J., Lyu J. (2020). Buttermilk as a wall material for microencapsulation of omega-3 oils by spray drying. LWT Food Sci. Technol..

[30] Chen I.S., Shen C.S.J., Sheppard A.J. (1981). Comparison of Methylene Chloride and Chloroform for the Extraction of Fats from Food Products. J. Am. Oil Chem. Soc..

[31] Singh H., Kumar C., Singh N., Paul S., Jain S.K. (2018). Nanoencapsulation of Docosahexaenoic Acid (DHA) Using a Combination of Food Grade Polymeric Wall Materials and Its Application for Improvement in Bioavailability and Oxidative Stability. Food Funct..

[32] Jiménez-Martín E., Gharsallaoui A., Pérez-Palacios T., Carrascal J.R., Rojas T.A. (2015). Volatile compounds and physicochemical characteristics during storage of microcapsules from different fish oil emulsions. Food Bioprod. Process..

[33] Zhu Y., Chen J., Chen X., Chen D., Deng S. (2020). Use of Relative Odor Activity Value (ROAV) to Link Aroma Profiles to Volatile Compounds: Application to Fresh and Dried Eel (*Muraenesox Cinereus*). Int. J. Food Prop..

[34] Mahdi A.A., Mohammed J.K., Al-Ansi W., Ghaleb A.D., Al-Maqtari Q.A., Ma M., Ahmed M.I., Wang H. (2020). Microencapsulation of fingered citron extract with gum arabic, modified starch, whey protein, and maltodextrin using spray drying. Int. J. Biol. Macromol. Struct. Funct. Interact..

[35] Kwamman Y., Klinkesorn U. (2015). Influence of Oil Load and Maltodextrin Concentration on Properties of Tuna Oil Microcapsules Encapsulated in Two-Layer Membrane. Dry. Technol..

[36] Ribeiro A.M., Estevinho B.N., Rocha F. (2021). The progress and application of vitamin E encapsulation—A review. Food Hydrocoll..

[37] Charles A.L., Abdillah A.A., Saraswati Y.R., Sridhar K., Balderamos C., Masithah E.D., Alamsjah M.A. (2021). Characterization of freeze-dried microencapsulation tuna fish oil with arrowroot starch and maltodextrin. Food Hydrocoll..

[38] Sun X., Cameron R.G., Bai J. (2019). Microencapsulation and antimicrobial activity of carvacrol in a pectin-alginate matrix. Food Hydrocoll..

[39] Edris A.E., Kalemba D., Adamiec J., Piątkowski M. (2016). Microencapsulation of Nigella sativa oleoresin by spray drying for food and nutraceutical applications. Food Chem..

[40] Bhusari S., Muzaffar K., Kumar P. (2014). Effect of carrier agents on physical and microstructural properties of spray dried tamarind pulp powder. Powder Technol..

[41] Cevik K., Yalcin H., Konca Y. (2024). Elucidating the Influence of Coating Materials in the Microencapsulation Process of Hempseed Oil Via Spray Drying: A Comprehensive Analysis of Physicochemical Attributes, Oxidation Stability, and Thermal Properties. Food Biophys..

[42] Botrel D.A., Fernandes R.V.d.B., Borges S.V., Yoshida M.I. (2014). Influence of Wall Matrix Systems on the Properties of Spray-Dried Microparticles Containing Fish Oil. Food. Res. Int..

[43] Maqsoudlou A., Mahoonak A.S., Mohebodini H., Koushki V. (2020). Stability and structural properties of bee pollen protein hydrolysate microencapsulated using maltodextrin and whey protein concentrate. Heliyon.

[44] Bajac J., Nikolovski B., Lončarević I., Petrović J., Bajac B., Đurović S., Petrović L. (2022). Microencapsulation of juniper berry essential oil (*Juniperus communis* L.) by spray drying: Microcapsule characterization and release kinetics of the oil. Food Hydrocoll..

[45] Kolanowski W., Jaworska D., Weißbrodt J., Kunz B. (2006). Sensory Assessment of Microencapsulated Fish Oil Powder. J. Am. Oil Chem. Soc..

[46] Steele R. (2004). Understanding and Measuring the Shelf-Life of Food.

[47] Sawitree S., Qixin Z., Benjawan T., Dudsadee U., Chureerat P., Savitri V., Vilai R. (2022). Optimization of Wall Material Composition for Production of Spray-dried Sacha Inchi Oil Microcapsules with Desirable Physicochemical Properties. Food Bioprocess Technol..

[48] Venkateshwarlu G., Let M.B., Meyer A.S., Jacobsen C. (2004). Modeling the sensory impact of defined combinations of volatile lipid oxidation products on fishy and metallic off-flavors. J. Agric. Food Chem..

[49] Karahadian C., Lindsay R.C. (1989). Evaluation of compounds contributing characterizing fishy flavors in fish oils. J. Am. Oil Chem. Soc..

[50] Ogrodowska D., Laaksonen O., Tanska M., Konopka I., Linderborg K.M. (2020). Pumpkin oil addition and encapsulation process as methods to improve oxidative stability of fish oil. LWT.

[51] Bi J., Lin Z., Li Y., Chen F., Liu S., Li C. (2021). Effects of different cooking methods on volatile flavor compounds of chicken breast. J. Food Biochem..

[52] Li Q., Li B., Zhang C., Zhou X., Liu W., Mi Y., Xie Z., Li Y., Li J. (2024). Insights into key aroma of vine tea (*Ampelopsis grossedentata*) for grade evaluation integrating relative odor activity value, gas chromatography-olfactometry and chemometrics approaches. Food Control.

[53] Gemert V. (1992). A Compilation of Odour, Flavour and Taste Threshold Values in Air, Water and Other Media. Chem. Senses.

